# Editorial: Integrating health psychology in practice: enhancing well-being and improving health outcomes across diverse contexts

**DOI:** 10.3389/fpsyg.2026.1930903

**Published:** 2026-07-29

**Authors:** Federica Mauro, Paul Greenman, Michela Di Trani

**Affiliations:** 1 Faculty of Medicine and Psychology, Sapienza University of Rome, Rome, Italy; 2 Département de Psychoéducation et de Psychologie, Université du Québéc en Outaouais, Gatineau, QC, Canada

**Keywords:** biopsychosocial model, context-sensitive care, contextual health, contextual intervention, health promotion, health psychology, person-centered care, psychological wellbeing

Health Psychology has progressively established itself as a key discipline for understanding health and illness through the lens of Engel's biopsychosocial model (Engel, 1977, 1980). Building on this framework, the discipline has evolved to examine how biological, psychological, behavioral, and social factors interact to influence health across the lifespan (Taylor, 2023; Marks et al., 2021). By recognizing that biological, psychological, and social dimensions continuously interact throughout the health–illness continuum, this perspective has expanded the concept of care beyond disease treatment, emphasizing health promotion, empowerment, participation, and person-centered care (World Health Organization, 1986). Rather than viewing health as a static endpoint, the biopsychosocial perspective highlights the dynamic interactions among biological, psychological, and social dimensions throughout the health–illness continuum. Such an approach encourages greater attention to the contexts in which health is experienced and promoted, recognizing the role of interpersonal relationships, organizational environments, and community resources in shaping health and wellbeing (World Health Organization, 1986, 2016). Within increasingly complex healthcare systems, Health Psychology is therefore called upon not only to develop effective interventions, but also to contribute to integrated and context-sensitive models of care capable of addressing the evolving needs of individuals, professionals, and communities.

The contributions collected in this Research Topic reflect the breadth and diversity of contemporary Health Psychology, emphasizing both theoretical developments and empirical evidence on the role of psychological processes in promoting health and wellbeing. Together, they illustrate how psychological knowledge can inform clinical practice, healthcare organization, and public health strategies while addressing the evolving needs of people across healthcare, educational, workplace, and community

settings. Collectively, the contributions can be grouped into five broad thematic areas, each addressing a distinct yet complementary dimension of psychological care, health promotion, and wellbeing.

The first group of contributions focuses on *patient-centered care, communication, and empowerment*, emphasizing the central role of trust, effective communication, and active patient participation in healthcare processes. Gabay highlights how trust is shaped by the quality of patient–provider communication, identifying key communication gaps—such as insufficient emotional support, limited patient involvement, and unclear information—that can undermine therapeutic relationships. Complementing this relational perspective, Varela et al. critically examine the concept of patient empowerment and propose a clearer conceptual framework, defining it as a dynamic process through which individuals develop the knowledge, skills, autonomy, and confidence to actively manage their health and participate in healthcare decisions. Finally, Loughnane et al. introduce Positive Health Coaching as a person-centered approach that integrates positive psychology, health psychology, lifestyle medicine, and coaching psychology to foster resilience, self-efficacy, and sustainable behavior change. Together, these contributions underscore that effective healthcare extends beyond clinical treatment to encompass trust, meaningful communication, empowerment, and the promotion of patients' active engagement in their own care.

A second thematic area examines *psychological interventions across healthcare settings*, presenting studies that evaluate evidence-based interventions designed to improve psychological adjustment, quality of life, and rehabilitation outcomes among individuals living with chronic diseases, mental health conditions, or other complex healthcare needs, underscoring the growing integration of evidence-based psychological approaches within multidisciplinary models of care. Diéguez et al. investigate the feasibility of Acceptance and Commitment Therapy (ACT) for patients with chronic kidney disease undergoing hemodialysis, providing preliminary evidence that ACT may reduce depressive symptoms and improve psychological flexibility in this vulnerable population. Lu and Xiao evaluate a structured group psychoeducation program for adults with major depressive disorder, demonstrating its effectiveness in enhancing benefit finding, while suggesting that longer interventions may be required to produce broader improvements in psychological wellbeing and depressive symptoms. In the field of dementia care, Domenicucci et al. compare standard Cognitive Stimulation Therapy with an adapted collaborative version, showing that both interventions improve psychosocial outcomes, with the collaborative approach yielding greater benefits in reducing social loneliness and strengthening socioemotional skills. Focusing on cardiovascular care, Tulloch et al. describe the rationale and design of *Healing Hearts Together*, an emotionally focused intervention aimed at improving relationship functioning and dyadic coping among couples following a cardiac event, whereas Crepaldi et al. demonstrate that integrating individualized psychological counseling into cardiac rehabilitation significantly reduces anxiety and depression while promoting a healthier perception of illness. Extending psychological care beyond medical settings, Craig et al. show that university counseling services effectively improve students' psychological wellbeing and reduce emotional distress, supporting the preventive role of psychological interventions during emerging adulthood. Finally, Zhou et al. synthesize the available evidence on non-pharmacological interventions for fear of childbirth, concluding that several psychological and behavioral approaches represent promising strategies for reducing childbirth-related fear and promoting maternal mental health. Collectively, these studies demonstrate the versatility of psychological interventions across diverse clinical and community settings, reinforcing their contribution to improving psychological adjustment, wellbeing, and health outcomes throughout the continuum of care.

The third thematic area explores *psychosocial adaptation to illness*, pointing to how psychological, relational, and social factors influence individuals' adjustment to acute and chronic health conditions. Sztajerowski et al. showed that attachment insecurity is associated with poorer mental health among couples facing cardiovascular disease, with common dyadic coping playing an important role in the adaptation process. Chandeying and Thongseiratch examined fear of cancer progression in gynecologic cancer survivor–caregiver dyads, demonstrating that greater fear is associated with poorer quality of life for both survivors and caregivers, emphasizing the need for dyadic approaches in psycho-oncology. Focusing on critically ill patients, Li, Yuan, et al. identified distinct PTSD profiles among mechanically ventilated ICU survivors, highlighting the importance of early psychological screening and tailored interventions following intensive care. Several contributions further explore the psychosocial burden associated with chronic illness and stigma. Li, Yang, et al. investigated the relationship between perceived stigma and depressive symptoms in patients undergoing maintenance hemodialysis, identifying stigma internalization and diminished self-worth as key mechanisms linking stigma to depression. In women with breast cancer, Chen et al. demonstrated that participation in ritual interactive activities fosters perceived emotional synchrony, which in turn reduces health information avoidance by enhancing positive emotions and coping self-efficacy. Stigma also emerged as a central issue in other clinical populations. Wang et al. reported a high global prevalence of stigma among individuals with schizophrenia through a systematic review and meta-analysis, while Zhang et al. qualitatively described the stigma experienced by stroke survivors and the coping strategies adopted to facilitate psychosocial adjustment. Finally, Zhao et al. documented a high prevalence of anxiety and depression among older adults with chronic diseases, identifying poor health status, limited social support, and functional impairment as major psychological risk factors. Taken together, these contributions demonstrate that successful adaptation to illness extends beyond medical management and requires addressing emotional distress, interpersonal relationships, stigma, and psychosocial resources throughout the disease trajectory.

Extending the focus beyond healthcare contexts, another group of contributions explores *health promotion and psychological wellbeing across educational, occupational, and community settings*, foregrounding the role of personal, relational, and environmental resources in fostering resilience and mental health. Zhan demonstrated that regular physical activity strengthens the relationships between self-efficacy, resilience, happiness, and wellbeing among young people with hearing disabilities, while Mieziene et al. revealing the reciprocal associations between psychological wellbeing, motivation, family support, and physical activity in adolescents. Together, these studies reinforce the importance of promoting healthy lifestyles as protective factors for psychological health across the lifespan.

The Research Topic also addresses wellbeing from educational and occupational perspectives, highlighting the influence of both personal and contextual factors. Li, Wang, et al. showed that psychological capital mediates the relationship between social support and employment anxiety among preschool teacher trainees, with professional identity strengthening this protective effect. In the workplace, Tong et al. found that effort–reward imbalance negatively affects emergency nurses‘ health through work–family conflict, whereas Renzi et al. explored aviation crew members' representations of wellbeing, emphasizing the influence of organizational culture, professional identity, and interpersonal relationships on occupational health.

Other contributions extend health promotion to broader developmental and relational contexts. Su demonstrated that memorable tourism experiences can enhance university students' sense of meaning in life by fostering self-expansion and psychological capital, whereas Yang et al. showed that both individual and partner self-esteem contribute to relationship satisfaction among first-year university students, underscoring the importance of interpersonal resources for wellbeing. Overall, these findings underscore the multifaceted nature of wellbeing, emphasizing how experiential, intrapersonal, and relational dimensions jointly contribute to positive developmental outcomes across different life domains.

The final thematic area brings together contributions that *strengthen the conceptual and methodological foundations of Health Psychology*, illustrating the importance of robust theoretical frameworks and reliable assessment tools for advancing research and practice. Mauro, Greenman, et al. propose a conceptual rethinking of health promotion grounded in the biopsychosocial model, arguing that health should be understood as a dynamic and context-dependent process emerging from the continuous interaction between individuals and their biological, psychological, social, and environmental ecosystems. Their perspective reinforces the need for person-centered and context-sensitive approaches that support individuals' capacity to adapt and actively engage with their health.

Several contributions focus on the development and validation of psychological assessment instruments. Guan et al. provide evidence for the longitudinal stability and reliability of the Edinburgh Postnatal Depression Scale among Chinese perinatal women, supporting its use for monitoring depressive symptoms across the perinatal period. Zhang et al. report encouraging psychometric properties for the Adolescent Story Stem Profile, highlighting its potential as a narrative-based measure of adolescents' socioemotional functioning and attachment representations. Likewise, Mauro, Di Trani, et al. validate the Italian version of the Psychologically Rich Life Questionnaire, demonstrating its sound psychometric properties and its associations with mindfulness, self-compassion, and cognitive fusion, thereby expanding the assessment of positive psychological functioning. Finally, Szulevicz and Arnfred discuss the opportunities and challenges associated with the use of the Strengths and Difficulties Questionnaire in educational psychology, emphasizing the need to balance standardized assessment procedures with context-sensitive professional judgment.

These contributions reinforce the advancement of Health Psychology by integrating conceptual innovation with methodological rigor, providing both theoretical perspectives and validated tools to support research, assessment, and evidence-based practice across diverse healthcare and educational contexts.

The contributions gathered in this Research Topic illustrate the breadth and vitality of contemporary Health Psychology, demonstrating its capacity to address health-related challenges across clinical, educational, occupational, and community settings. Although diverse in their theoretical perspectives, methodologies, and target populations, the studies share a common commitment to advancing a biopsychosocial understanding of health that integrates psychological processes with relational, organizational, and contextual dimensions of care. Collectively, they shed light on the importance of promoting person-centered, evidence-informed, and context-sensitive approaches that not only support individuals in managing illness, but also foster wellbeing, resilience, and active participation throughout the lifespan. We hope that this Research Topic will stimulate further interdisciplinary research and encourage the continued development of innovative psychological models and interventions capable of responding to the evolving health needs of contemporary societies.
